# The Organisation of Military Medical Services

**Published:** 1915-07-24

**Authors:** 


### The Hospital

The Workers' Newspaper of Administrative Medicine and Institutional Life, Administration, National Insurance and Health.

No. 1519, Vol. LVIII. SATURDAY, JULY 24, 1915. PRICE ONE PENNY

### THE ORGANISATION OF MILITARY MEDICAL SERVICES.

The need for the concentration and organisation of the total capacity of the nation in order to bring the war to a satisfactory termination is now widely recognised. The struggle, it is evident, can hope for no early ending, and the task is clearly beyond the competence of mere official persons. It is a question of national existence, and by national effort alone will the problem be brought to victorious solution. The arrangements which are now being made to take note of the forces available in order that these may be effectively adjusted and utilised can hardly fail to appeal to members of the medical profession. These by their training and experience have learned how blind may be uninformed individual effort, and how essential to success in many departments of activity are co-ordination and plan. Especially, of course, is this true when expedition is a pressing element and when time forbids that we should wait for the uncertain appearance of personal inspiration and individual initiative. Not that these have not their place and function. Quite the contrary, indeed, but organisation does not necessarily destroy them, and there are even minds which only become fully awake to their own capacities under the stimulus provided by union with others for a common purpose. This state of mental activity and of co-operation for practical purposes is just now much to be desired, for all other issues have become of subordinate importance in the face of the supreme question of the hour. It is to this end that every individual citizen's power of clear and competent thinking ought to be directed.

An example of what can be done by the organised study of difficult and complex problems is the success which has attended the efforts made to protect our fighting forces from disease. Nowhere has there been less room for criticism, and the contrast presented by the present war, as compared with former wars, has, in this respect, been most conspicuous. The explanation doubtless is that questions of hygiene, sanitation, and public health have now been for some years studied by a scientifically organised medical service, and that all contributions from individuals have been gathered into a common body of doctrine and practice. The service from the very nature of its functions has, at least in some measure, operated under the inspection of public bodies. It has thus had the advantage of lay co-operation and criticism, and has been kept in contact with the actualities of national life. Doubtless sometimes this criticism has been wanting in comprehension and sympathy, but on the whole it has been advantageous if only in bringing to the questions of the hour judgments having a wider outlook than those which can be professed by technical workers and experts. It is from this scheme of well-balanced organisation that have arisen the knowledge and capacity which, applied to the sanitary problems created by armies in the field, have saved our fighting men in Flanders from the disasters which in bygone days only too often fell upon others engaged in similar enterprises. It seems a plain lesson that to obtain equally satisfactory results in other directions a similar policy must be inaugurated. That forces of value are at present lying concealed and unfruitful there can be no moral doubt, and it is because of this that the summons to the nation to bend all its energies to the common cause has an inspiring and encouraging outlook. The official world must be taught that it has no monopoly of competence and insight; indeed, its immediate business is to discover these qualities wherever they exist and to afford them opportunities for national service. To take a single and somewhat limited example, it seems plain from the public prints that even the Royal Society has need to learn this lesson. The protests in the world of science excited by the methods of the Council of the Society are very similar to those which were heard in a larger world in the early months of the war. Natural though it may be for officials to think that with them is all knowledge, the present circumstances demand that this pleasant delusion shall be dissipated.

To a large extent it may be readily admitted that the necessity in the existing emergency for the breaking down of formal barriers has been widely recognised in the military service of the Army.

Possibly some may even say that the practice has been carried to such an extent as to prejudice the welfare of the civil population. However that may be, there is abundant evidence that the medical profession has shown itself fully awake to the peculiar needs of the hour and not a few heavy sacrifices have been made by individuals to help to satisfy these. This is not to say that everything necessary has been done and that we may rest on our oars. Far from it; we are fully convinced, indeed, from some attention given to the subject, that there is yet much work which the profession is able and ready to undertake, provided only official obstacles are taken out of the way. Light will no doubt be thrown on this subject by the information afforded by the Registration Act and by the efforts which are now being made to secure more doctors for service with the new Armies. In our issue of July 10 we directed attention to the work being undertaken with this end by a national Scottish Committee, and we are glad to know that a very similar scheme has been drafted by the War Emergency Committee organised by the British Medical Association for the Metropolitan area. This latter deserves very grateful recognition for the census and other work it has performed, but we should like to see it developed on a somewhat broader basis. There have been so many contentious professional questions of recent years that it may be doubted whether any medico-political organisation is a suitable medium for the organisation of the profession for a national emergency. We are far from forgetting that the present Committee took up the task when no one else seemed willing to move, and for this we desire to give them every credit. If they can now enlarge the scope of their appeal they will probably find willing helpers, and we are sure that nothing in the shape of a narrow jealousy will hinder them from promoting the fullest possible success of the cause to which they have set their hands.

